# Environmental epimutagens, epigenetic memory, and cancer latency

**DOI:** 10.1093/narcan/zcag033

**Published:** 2026-11-11

**Authors:** Paola Arimondo, Xavier Coumoul, Johanna Lepeule, Marie Lopez, Charlotte Proudhon, Laurent M Sachs, Pierre-Antoine Defossez

**Affiliations:** Epigenetic Chemical Biology EpiCBio, Institut Pasteur, CNRS UMR3523 Chem4Life, Université de Paris Cité, 28 rue du Dr Roux, 75015 Paris, France; “France Environmental Epigenetics” Network, 75013 Paris, France; “France Environmental Epigenetics” Network, 75013 Paris, France; Université Paris Cité, INSERM, Health & Functional Exposomics, HealthFex, 75006 Paris, France; “France Environmental Epigenetics” Network, 75013 Paris, France; Université Grenoble Alpes, INSERM U1209, CNRS UMR5309, Institut pour l'Avancée des Biosciences (IAB), 38000 Grenoble, France; “France Environmental Epigenetics” Network, 75013 Paris, France; Institut des Biomolécules Max Mousseron (IBMM), UMR5247 CNRS-UM-ENSCM, 1919 route de Mende, 34090 Montpellier Cedex 5, France; “France Environmental Epigenetics” Network, 75013 Paris, France; University of Rennes, INSERM, EHESP, Irset (Institut de Recherche en Santé, Environnement et Travail)—UMR_S 1085, 35000 Rennes, France; “France Environmental Epigenetics” Network, 75013 Paris, France; Centre National de la Recherche Scientifique and Muséum National d’Histoire Naturelle, UMR 7221 Physiologie Moléculaire et Adaptation, Département Adaptation du Vivant, Alliance Sorbonne Universités, CP32 57 rue Cuvier, 75231 Paris, Cedex 5, France; “France Environmental Epigenetics” Network, 75013 Paris, France; Université Paris Cité, CNRS, Epigenetics and Cell Fate, 75013 Paris, France

## Abstract

Environmental exposures can cause cancer through DNA damage and mutations, but whether they can also leave durable, cancer-relevant changes in gene regulation is less clear. Chromatin helps maintain cell identity, differentiation state, and plasticity, providing a route by which non-genetic effects of exposure could persist after exposure. We define environmental epimutagens as agents or conditions that perturb chromatin regulation, and examine how such perturbations may influence cancer initiation and latency. Exposures can reach chromatin through altered metabolite or cofactor availability, interference with chromatin-regulating proteins, and direct chemical modification. Most resulting changes are transient, adaptive, or secondary. The central question is which become biologically embedded in long-lived cell populations, remain silent during latency, and alter responses to later genetic, inflammatory, or tissue-level pressures. Studies of inflammatory memory and transient disruption of chromatin repression show that short-lived perturbations can durably reprogram cellular potential, lower the threshold for tumor initiation, or generate tumorigenic states. Exposures may also influence the later selection of primed cells. Establishing causality will require physiologically realistic exposure models, longitudinal and lineage-resolved analyses, and functional tests distinguishing biomarkers of exposure from persistent mediators of cancer susceptibility. Such work may identify preventable exposures, informative biomarkers, and opportunities for cancer interception.

## Graphical Abstract

**Graphical Abstract:**
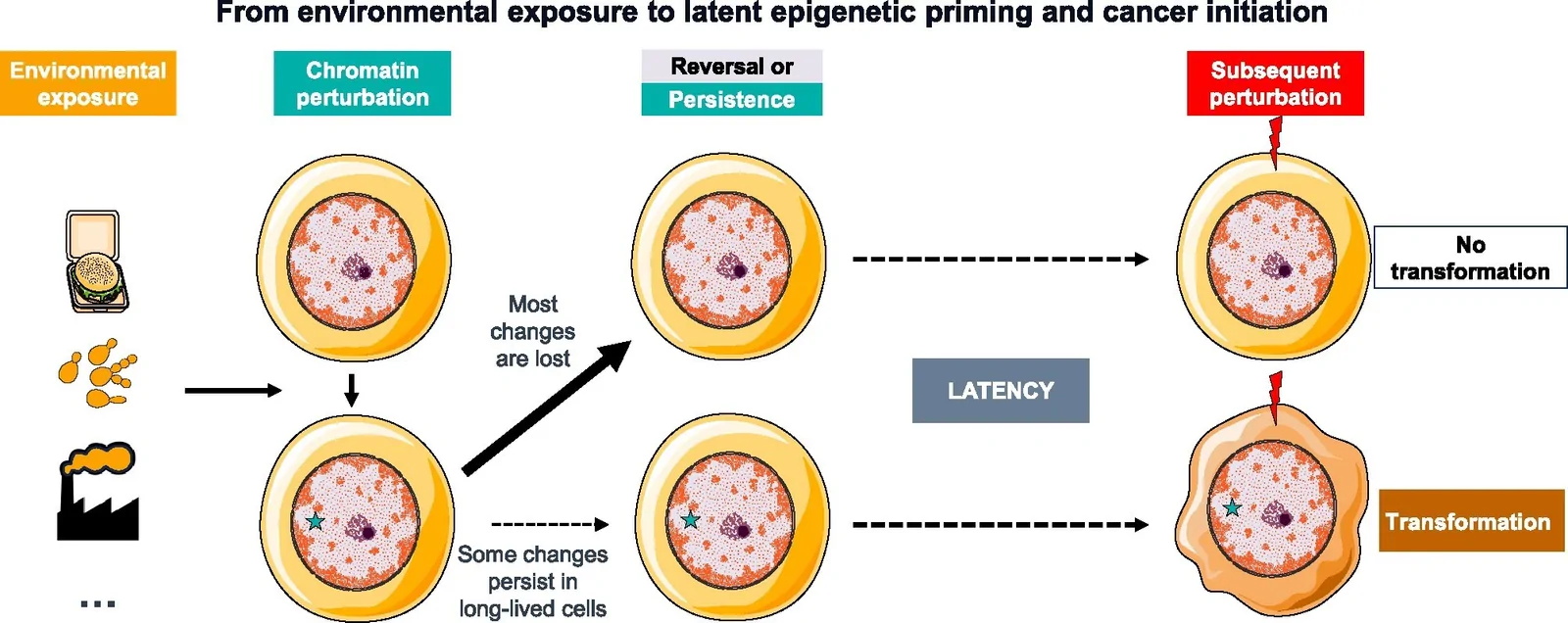

Impact statement Environmental epimutagens—agents that perturb chromatin without altering DNA sequence—potentially influence cancer risk long after exposure has ended. This short review explores how such changes could occur, how they could persist silently during cancer latency, and how they may increase the chance that affected cells develop into cancer. Understanding which epigenetic changes persist, which cells retain them, and how they influence cancer risk could improve the interpretation of biomarkers, help identify individuals or tissues at increased risk, and open up new opportunities for cancer prevention and interception before tumors develop.

## Introduction

The causal effect of environmental exposure on tumorigenesis was demonstrated more than a century ago, in the pioneering experiments of Katsusaburo Yamagiwa, who showed that coal tar painted on rabbit ears induced squamous cell carcinomas [1]. Today, understanding the links between environment and cancer risk is still a pressing concern. For instance, the incidence of several malignancies has risen markedly in younger adults over only a few decades, a timescale incompatible with genetic evolution and instead pointing toward changing environmental and lifestyle exposures [2]. Yet cancers often emerge long after the relevant exposure, raising a fundamental mechanistic question: how can an environmental perturbation leave a biological memory that influences cancer risk years later?

Cancer arises not only through genetic lesions, but also through durable changes in gene regulation that alter cell identity, differentiation state, plasticity, and therapeutic response [3, 4]. Chromatin is central to these processes: DNA methylation, histone modifications, nucleosome organization, and chromatin-associated regulators help establish and maintain transcriptional states through cell division [5, 6]. Replication-coupled mechanisms contribute to this continuity: UHRF1 and DNMT1 restore DNA methylation after replication, while recycled parental histones help re-establish local histone-modification patterns [7–9]. These mechanisms are less faithful than DNA sequence inheritance [10, 11], but allow regulatory information to persist through mitotic divisions. Altogether, these properties place chromatin at the interface between environmental exposure and cancer risk [12].

In this review, epigenetic refers broadly to chromatin-based regulation not encoded by a change in DNA sequence. We use the term environmental epimutagen operationally [13] to describe an environmental agent that perturbs chromatin regulation, focusing on perturbations relevant to cancer initiation, latency, and evolution. This definition does not require transgenerational inheritance, and does not imply that every exposure-induced chromatin change is stable or pathological. Indeed, many chromatin responses are transient or adaptive, and many epigenetic abnormalities observed in tumors are secondary to genetic lesions [14]. We use epigenetic memory for an exposure-induced alteration that persists after withdrawal of the initiating trigger. The key question is therefore whether environmentally induced chromatin changes persist and contribute to cancer phenotypes.

We first describe the overlapping levels of environmental effects that chromatin is exposed to. We then focus on mechanistic routes by which environmental exposures affect chromatin in mammalian cells, emphasizing metabolism, metal, and cofactor availability, and interference with chromatin-regulating proteins. We next discuss how initially reversible perturbations may become stabilized through cell-state transitions, clonal selection, or cooperation with genetic alterations. A central issue is whether such perturbations can become embedded in long-lived somatic stem or progenitor compartments, stay restrained during latency, and later promote clonal expansion under pressures associated with aging, inflammation, tissue injury, or additional exposures [15–17]. This framework may help distinguish transient exposure signatures from cancer-relevant epigenetic alterations and guide future efforts to connect environmental exposures with cancer mechanisms.

## Overlapping environmental contexts shape the cancer epigenome

Environmental effects on chromatin can arise at several levels (Fig. 1A). Cell-intrinsic factors include genotype, developmental state, cell-cycle position, and the pre-existing chromatin landscape, which together determine how a cell responds to a perturbation. The tissue microenvironment includes the extracellular matrix, oxygenation, neighboring stromal and immune cells, and paracrine signals. The organismal environment includes endocrine and metabolic state, inflammation, tissue stress, and circadian rhythms. Finally, the external environment includes diet, pollutants, toxicants, pathogens, and radiation [12, 18].

**Figure 1.**
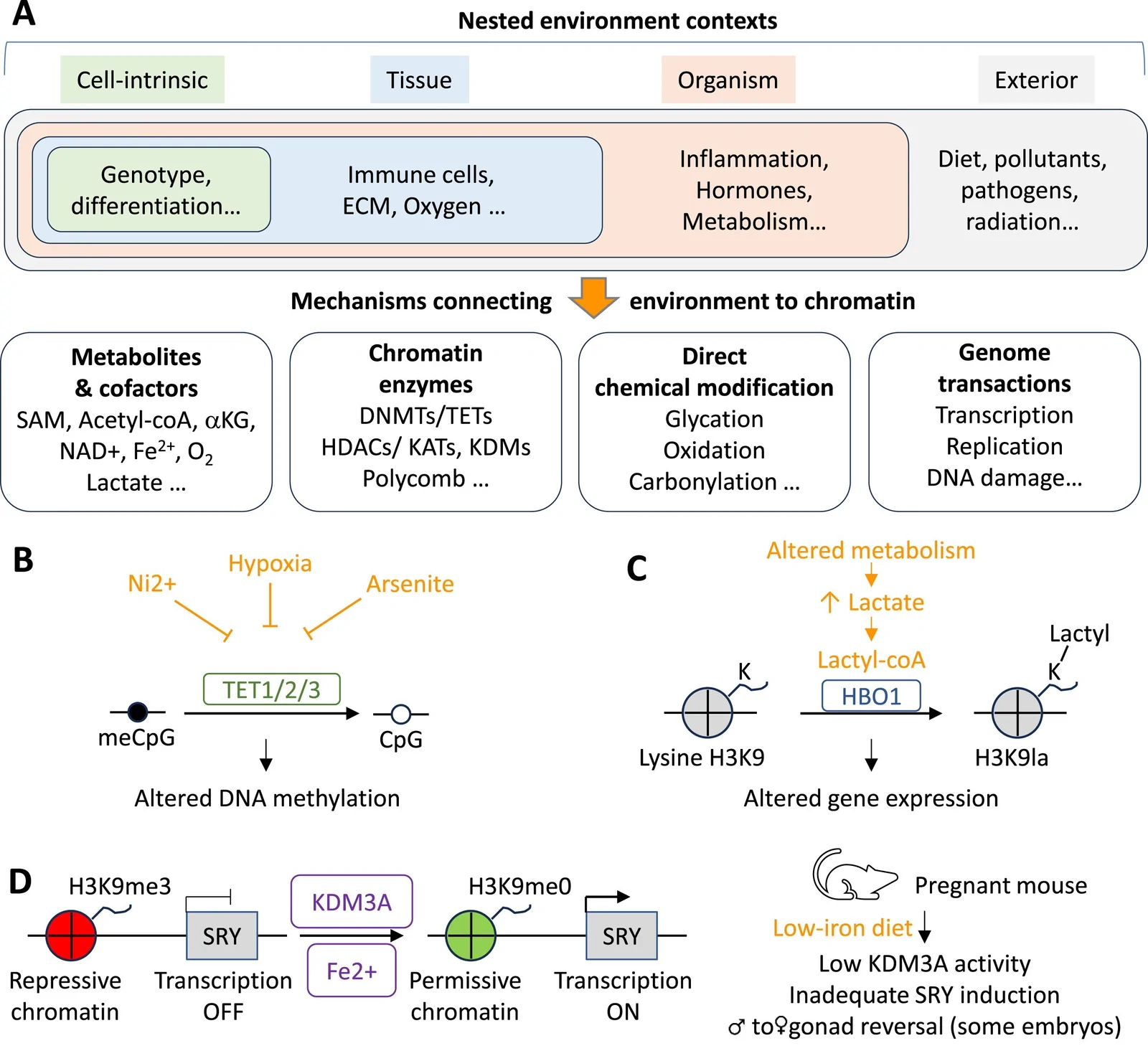
Environmental routes to chromatin disruption. (**A**) Environmental influences arise at multiple nested biological contexts, from the external environment to tissue, organismal, and cell-intrinsic factors. These contexts converge through several mechanistic routes that perturb chromatin regulation, including altered metabolite and cofactor availability, modulation of chromatin-modifying enzymes, direct chemical modification of chromatin components, and changes in genome and chromatin processes. (**B**) Environmental and tissue conditions can interfere with TET-dependent DNA demethylation. Nickel can displace the Fe²⁺ cofactor required for TET activity, severe hypoxia limits the availability of O₂ required for catalysis, and arsenite can inhibit TET proteins, thereby altering DNA methylation. (**C**) Altered metabolism can increase lactate and lactyl-CoA availability. HBO1 uses lactyl-CoA to catalyze H3K9 lactylation, which is associated with altered gene expression. (**D**) Physiological iron availability can regulate chromatin and cell fate *in vivo*. In mouse embryos, maternal iron deficiency reduces KDM3A activity and H3K9 demethylation at the Sry locus, impairing Sry induction and causing male-to-female gonadal sex reversal in a subset of embryos.

These categories overlap: an external exposure may first alter systemic metabolism, endocrine function, or inflammatory signaling; these changes may then remodel a tissue niche and ultimately affect chromatin regulation in a susceptible cell [19]. Conversely, the same exposure may produce different outcomes according to genotype, developmental stage, age, tissue context, dose, and duration [17, 18].

This complexity has key consequences for experimental design: simple cell culture may be appropriate when the exposure acts cell-autonomously, but it will miss effects mediated by tissue architecture, immune signaling, metabolism, or the microbiome [19, 20]. In addition, these considerations imply that environmental epimutagens should not be sought only among compounds that bind DNA or histones directly. Many could act indirectly, for instance by altering the biochemical environment of chromatin regulators, as we will now discuss.

## Molecular routes from environmental exposure to chromatin disruption

### Metabolic and cofactor control of chromatin regulation

Many chromatin-modifying enzymes use metabolites as substrates or cofactors, or are inhibited by metabolic products [21, 22]. As a result, environmental perturbations of metabolism can be transmitted to chromatin through changes in enzyme activity, possibly resulting in gene-regulatory alterations relevant to cancer (Fig. 1A).

DNA methylation illustrates this principle. DNA methyltransferases depend on S-adenosyl-methionine (SAM) as the methyl donor, linking one-carbon metabolism to DNA methylation potential [14, 23]. Nutritional or metabolic changes affecting folate, methionine, choline, betaine, serine, or glycine metabolism can alter SAM levels and the SAM:SAH ratio, thereby changing methylation potential [23–26]. Additional factors beyond total SAM abundance and SAM:SAH ratio also influence global genomic methylation: local metabolite availability, subcellular compartmentalization, recruitment and activity of the methyltransferases [27, 28], chromatin accessibility, and competition with other methylation reactions may also be rate-limiting. The TET enzymes, which catalyze cytosine oxidation during active DNA demethylation, depend on Fe²⁺ and α-ketoglutarate and are inhibited by metabolites such as succinate and 2-hydroxyglutarate [29]. Oxygen availability can itself be limiting: hypoxia is sufficient to reduce TET activity, decrease 5-hydroxymethylcytosine, and promote DNA hypermethylation in cultured cells and tumors [30]. These biochemical sensitivities constitute entry points by which environmental or metabolic perturbations can influence DNA methylation (Fig. 1B).

Recent work further supports the notion that histone modification depends not only on the total cellular concentration of a metabolite, but also on where it is produced. In the Drosophila wing disc, local production of nuclear acetyl-CoA by ACSS2 generates spatially restricted histone acetylation and affects developmental gene expression [31]. More broadly, native chromatome profiling has identified hundreds of metabolic enzymes associated with chromatin across healthy tissues and cancers, with marked tissue specificity [32]. Therefore, chromatin-associated metabolism creates local regulatory environments that cannot necessarily be predicted from bulk metabolite measurements, a finding that will need to be taken into account in future research.

There is clear evidence that pathological metabolite pools can impose cancer-relevant chromatin states. Succinate dehydrogenase-mutant paragangliomas accumulate succinate, while IDH-mutant malignancies produce 2-hydroxyglutarate, both causing a hypermethylator phenotype causally involved in tumorigenesis [33–36]. These examples are not environmental exposures per se, but they provide proof of principle that altered metabolite levels can reshape chromatin regulation, with effects on gene expression and cell transformation. They also explain the growing concern over SDH-inhibiting fungicides, which in cells were shown to reduce SDH activity, increase succinate, and induce a Warburg-like metabolic shift associated with enhanced migration and invasion, particularly in cells already carrying cancer-driver alterations [37]. However, we note that this study did not establish that chromatin remodeling mediates these phenotypes or that they occur at realistic human exposure levels. Moreover, genetic loss of SDHB and succinate accumulation are not by themselves sufficient for tumorigenesis, indicating that succinate is unlikely to provide a complete mechanistic explanation [38]. Thus, chemical inhibition of SDH may reproduce some features of SDH-deficient tumors, but direct evidence connecting environmental exposure, persistent chromatin alteration, and cancer initiation remains incomplete.

Acetyl-CoA and other acyl-CoA species influence histone acetylation, crotonylation, butyrylation, and other acyl marks, linking diet, microbiome activity, and cellular metabolism to chromatin regulation [39–41]. Lactate is particularly relevant in cancer because glycolytic tumors accumulate lactate, histone lactylation increases with glycolytic flux, and enzymes responsive to lactate have recently been linked to tumorigenic pathways, including p53 regulation [42–44]. Similarly, HBO1 was recently identified as a lactyltransferase that preferentially catalyzes H3K9 lactylation at transcription start sites; HBO1-dependent lactylation influenced transcriptional programs and tumor-associated phenotypes [45]. This provides a direct enzymatic connection between lactate metabolism and a defined histone modification, although the importance of this pathway for environmentally induced cancer initiation remains to be determined (Fig. 1C). Lactate also directly binds a number of proteins, including the corepressor TRIM28, modifying the transcription of its target genes [46].

Another striking report showed that the food preservative sodium benzoate directly increases lysine benzoylation in histones, a mark associated with gene expression [47]. It must be noted, however, that the concentration of sodium benzoate used in these studies is orders of magnitude greater than concentrations plausibly achieved through dietary exposure. Again, biochemical activity at high experimental concentrations does not imply that a compound perturbs chromatin under real-life conditions.

As for the TET enzymes, histone demethylases can be sensitive to oxygen availability. A recent paper using breast cancer cells showed that hypoxia reduced the activity of the H3K4me3 demethylase KDM5, which paralleled a shift of H3K4me3 peaks and a change of gene expression [48].

### Disruption of chromatin regulation by environmental metals

Environmental metals can perturb chromatin regulation through several mechanisms. Deficiency of an essential metal can limit the activity of chromatin enzymes; a toxic metal may displace its physiological cofactor; and thiol-reactive metals can bind cysteine-rich protein domains or act indirectly through altered metabolism and other pathways. The evidence for direct interference with a defined chromatin regulator is considerably stronger for some exposures than for others.

A recent paper showed that maternal iron deficiency impaired KDM3A-mediated H3K9 demethylation at the mouse Sry locus, suppressed Sry expression, and caused male-to-female sex reversal in genetically susceptible embryos (Fig. 1D) [49]. Admittedly, this is a developmental rather than a cancer model, yet it provides strong evidence that physiological metal availability can control a specific chromatin enzyme, gene expression, and cell-fate decisions *in vivo*. Nickel provides an example of direct interference, as Ni²⁺ can displace Fe²⁺ in TET enzymes and inhibit 5-methylcytosine oxidation [50].

Arsenic is another important environmental molecule to consider because it is an established human carcinogen with comparatively weak direct mutagenic activity [51] and has repeatedly been associated with changes in DNA methylation and histone modifications [52]. One potential direct molecular effect is that Arsenite binds the cysteine-rich Zinc fingers of TET enzymes and decreases their activity in biochemical assays [53]; whether this contributes to the effects seen in cells and organisms remains to be determined. Arsenic exposure has also been reported to cause a genome-wide gain of H3K27me3 and loss of H3K9me3 in human bronchial epithelial cells, associated with an altered balance between PRC2 complexes [54]. However, the molecular causes for this observation are unknown, and it is unclear whether these changes persist after exposure or play a role in arsenic-induced cancer in humans. While arsenic does impact chromatin, its effects are pleiotropic and also involve oxidative stress, altered metabolism, signaling, and DNA repair [55].

Similarly, cadmium and its derivatives are human carcinogens [56], and they likely act through various parallel mechanisms including altered epigenetic regulation, but also oxidative stress and impaired DNA repair [55]. As for Arsenic, it is therefore difficult to isolate chromatin disruption from the broader cellular stress response or to assign the carcinogenic effect to a single molecular pathway. Molecularly, cadmium can substitute for zinc in zinc finger transcription factors [57], which could potentially disrupt transcriptional equilibria or the function of certain methyl-CpG-binding proteins [58, 59], although this remains to be tested.

### Direct chemical modification of chromatin

Some exposures can affect chromatin directly, by covalently modifying DNA or histones [60]. Unlike canonical modifications, they are formed through chemical reactivity rather than the targeted action of a chromatin-modifying enzyme.

A well-described example is methylglyoxal, a reactive by-product of glycolysis, which modifies histone lysines and arginines, generating abundant non-enzymatic adducts that can interfere with canonical histone modifications [61]. Histone glycation can also alter chromatin architecture, and at least some of these modifications are reversible, suggesting that cells may possess mechanisms to limit or repair chemically induced chromatin damage [62]. Such mechanisms are particularly relevant to cancer because altered glucose metabolism, oxidative stress, and detoxification capacity may increase the burden of reactive metabolites in tumors.

### Chronic low-dose exposure and mixtures are challenging to study

To close this section, we wish to underline that a major issue of current research is the disconnect between experimental designs and human exposure. Mechanistic studies commonly examine one compound at a time, often at high concentrations selected to produce a measurable molecular response, whereas human exposures are generally low-level, chronic or intermittent, and involve mixtures whose composition and intensity vary over time [20]. Mixture effects cannot necessarily be predicted from the activity of their individual components. For example, endocrine-disrupting chemicals that have little or no activity individually at a given concentration can bind cooperatively and synergistically activate the pregnane X receptor when combined [63]. This provides a mechanistically understood example of a “cocktail effect,” although it does not demonstrate a persistent chromatin alteration or a role in cancer initiation. More broadly, multiple low-level exposures could converge on processes such as redox balance, one-carbon metabolism, metal and cofactor availability, mitochondrial function, or inflammatory signaling, even when no single agent produces a detectable chromatin effect.

At present, direct evidence that environmentally realistic mixtures produce cumulative chromatin alterations, that these changes persist after exposure withdrawal, and that they contribute to cancer initiation remains scarce; this constitutes a major knowledge gap for future efforts to address.

## From transient chromatin perturbation to stable epigenetic priming

### Most chromatin responses are reversible

Environmental stimuli can alter transcription-factor binding, histone modifications, chromatin accessibility, or DNA methylation, but most such changes disappear after exposure withdrawal (Fig. 2A). Indeed, chromatin-associated proteins and modifications turn over, while DNA replication and mitosis transiently disrupt or dilute many chromatin features [64]. Once the stimulus is removed, the pre-existing combination of DNA sequence, transcription factors, chromatin regulators, and extracellular signals generally re-establishes the original state. In addition, replication-coupled maintenance and feedback between chromatin marks tend to reconstruct the state favored by the remaining regulatory context [10]. Furthermore, in tissues, short-lived cells turn over, an additional way by which exposure-induced alterations can disappear. Exposure-induced chromatin changes should therefore not automatically be equated with stable “epimutations.”

**Figure 2.**
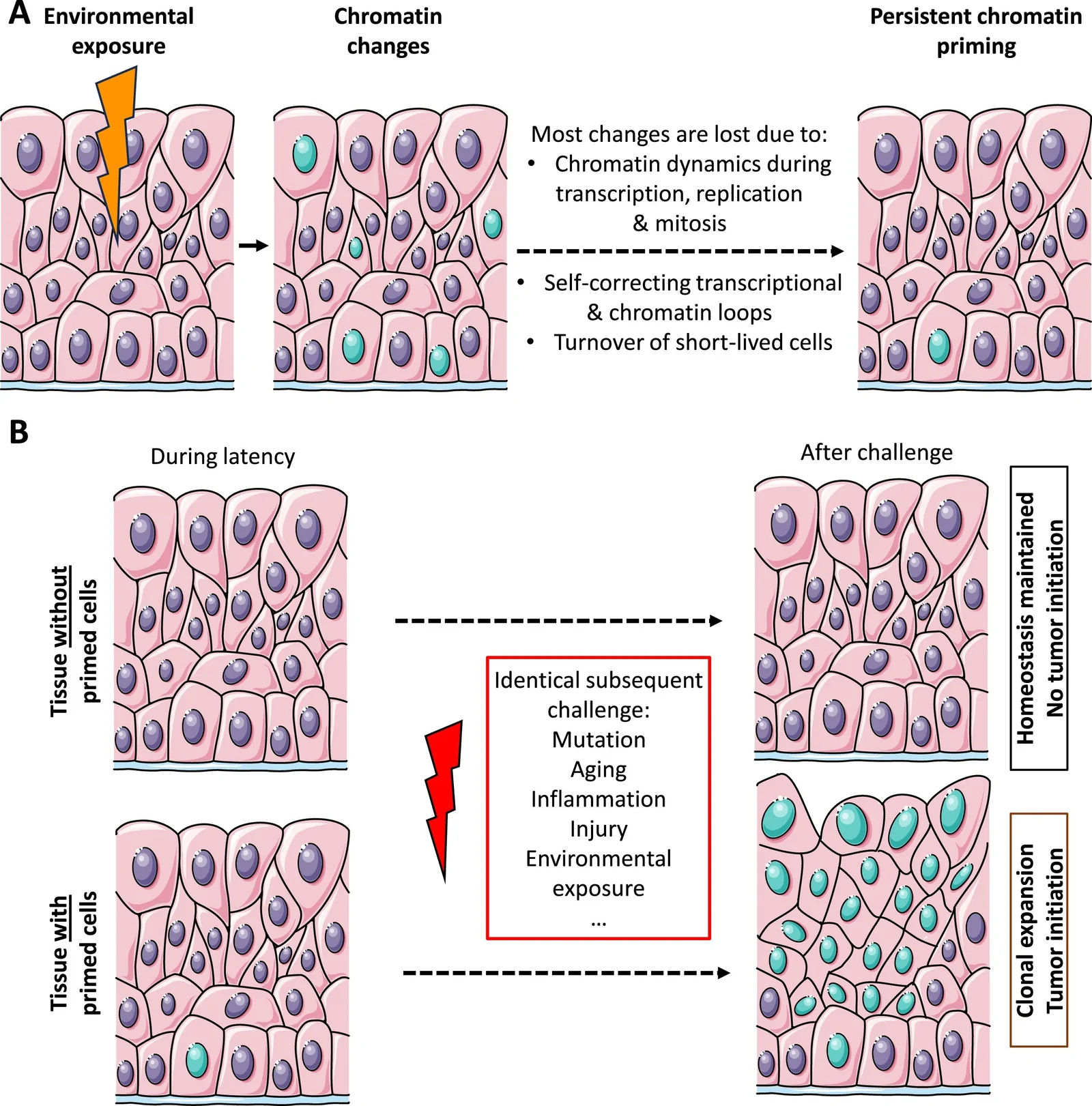
Persistence of exposure-induced chromatin states and their potential contribution to tumor initiation. (**A**) Environmental exposure induces chromatin alterations in multiple cells within a tissue. Most alterations are subsequently lost through various mechanisms. Rare alterations may nevertheless persist in long-lived cells, producing a state of persistent chromatin priming. Violet nuclei: unaltered, turquoise nuclei carry an exposure-induced chromatin alteration. (**B**) Model illustrating how persistent priming may influence the response to a later challenge. During latency, tissues containing a primed cell may remain phenotypically normal. A subsequent challenge, such as an oncogenic mutation, aging, inflammation, tissue injury and regeneration, or environmental re-exposure, does not necessarily induce tumor formation in unprimed tissue. In persistently primed tissue, however, the same challenge may confer a selective advantage on the primed cell and its descendants, promoting clonal expansion and tumor initiation. This model does not imply that persistent priming is sufficient for transformation, but that it may alter susceptibility to later tumor-promoting events.

### Some alterations can persist after the disturbance is removed

Nevertheless, some chemically induced changes have been shown to persist after the initiating stimulus was removed. For instance, in mouse embryonic stem cells, a short pulse of the histone deacetylase inhibitor trichostatin A caused widespread changes in transcription, histone modifications, and genome organization [65]. Most of the changes reversed after drug withdrawal, but a subset of genes retained altered expression and three-dimensional contacts, and a second exposure produced more persistent transcriptional deregulation [65]. Thus, a transient chromatin perturbation can leave a selective regulatory memory rather than causing either complete restoration or uniform persistence.

Ideally, a single study would establish the entire sequence from a realistic exposure to a persistent chromatin alteration in a defined cellular reservoir and, after a period of latency, to altered cancer susceptibility, while testing causality along the way (Fig. 3A). In practice, few studies meet this stringent standard: most provide valuable evidence, but for only part of the chain, as we shall now illustrate with the examples below, summarized in Fig. 3B.

**Figure 3.**
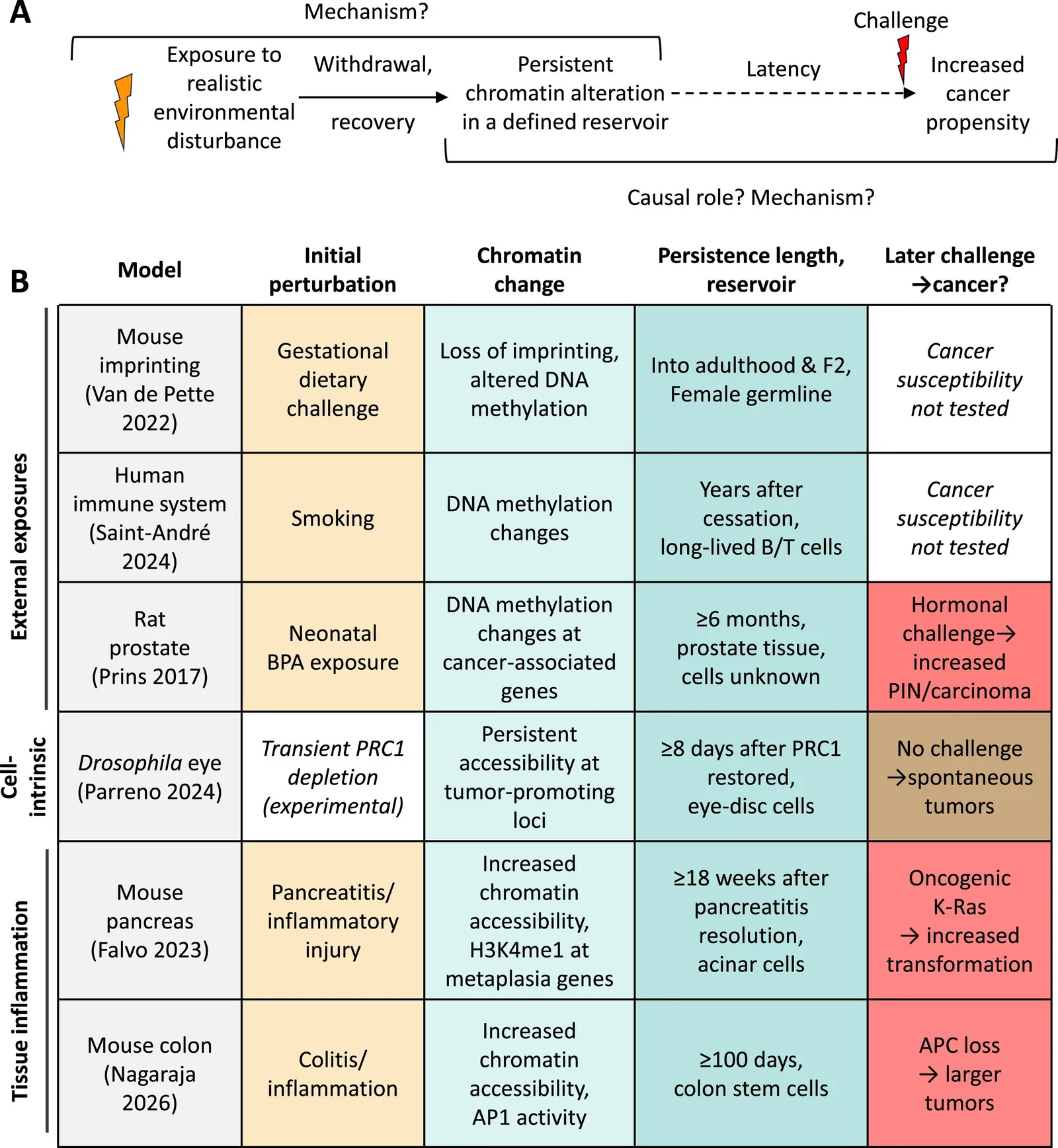
Experimental framework and current evidence for persistent epigenetic priming in cancer. (**A**) Framework for testing whether an environmental perturbation induces a persistent chromatin alteration that modifies later cancer susceptibility. Exposure withdrawal, demonstration of persistence in a defined cellular reservoir, and a subsequent challenge distinguish transient effects from latent priming; mechanistic intervention is required to establish causality. (**B**) Selected studies illustrating complementary parts of this framework. External exposures provide evidence for persistent epigenetic effects, with developmental BPA exposure additionally linked to increased later cancer susceptibility. Cell-intrinsic PRC1 depletion and inflammatory tissue models provide more direct evidence that persistent regulatory states can promote tumorigenesis or alter responses to subsequent oncogenic events.

Imprinted genes are monoallelically expressed, according to parental origin, and their regulation is chromatin-based, involving DNA methylation and histone modifications [66]. They frequently control growth and are often misexpressed in cancer, where they may be protumoral [67]. Several interesting papers have shown that environmental perturbations, for instance pollution by Bisphenol-A [68] or adverse nutritional conditions *in utero* [69, 70], lastingly affect the expression of imprinted genes. It remains to be tested whether this translates into higher propensity for subsequent tumorigenesis.

Evidence for persistent exposure effects also exists in humans. In the Milieu Intérieur cohort, cigarette smoking was associated with changes in adaptive immune responses that remained detectable years after smoking cessation and correlated with persistent DNA hypomethylation at various loci [71]. These findings do not establish that the methylation changes themselves mediate cancer susceptibility, but they constitute an example in humans in which a real-world environmental exposure is associated with durable epigenetic and functional effects after exposure cessation.

Some studies successfully bridge a transient environmental exposure to later cancer risk. For instance, in rats, neonatal exposure to bisphenol A induced DNA methylation changes at cancer-associated genes in the prostate that remained detectable >6 months after exposure. Developmental BPA exposure also increased prostatic intraepithelial neoplasia and carcinoma following a later hormonal challenge [72]. The cellular reservoir of the persistent changes and the causal contribution of DNA methylation to increased cancer susceptibility were not established, but this model links a transient environmental exposure to persistent epigenetic alteration and an enhanced response to a later cancer-promoting challenge.

### From persistent regulatory memory to a cancer cell state

The examples above establish that transient environmental perturbations can leave a regulatory memory and, in some cases, are associated with increased later cancer susceptibility. Earlier human studies provide important precedent for malignant states that depend strongly on epigenetic regulation in the absence of recurrent genetic changes that readily explain the phenotype. In posterior fossa group A ependymoma, Mack *et al*. identified tumors with very low mutation burden but a distinctive CpG island methylator phenotype and Polycomb-associated repression [73]; Michealraj *et al*. later linked this epigenetic state to the hypoxic developmental microenvironment and altered H3K27 regulation [74]. In pancreatic ductal adenocarcinoma, McDonald *et al*. showed that distant metastases undergo large-scale chromatin reprogramming coupled to altered glucose metabolism [75], while Makohon-Moore *et al*. found that known driver mutations were largely shared among metastatic lesions rather than being metastasis-specific [76]. These studies do not recapitulate an exposure-withdrawal-latency sequence, but they offer compelling evidence for metabolic or microenvironmental control of cancer-relevant epigenetic states. Against this background, Parreno and colleagues provided a particularly direct proof of principle that a transient chromatin perturbation can itself initiate a stable tumorigenic state [77]. Transient and reversible depletion of Polycomb repressive complex 1 components in the developing Drosophila eye induced tumors that persisted after normal Polycomb protein levels were restored, without evidence of recurrent genetic driver alterations. The malignant state was associated with irreversible activation of tumor-promoting genes, including components of the JAK–STAT pathway and *zfh1*, the fly homolog of the human *ZEB1* oncogene [77]. This developmental model does not involve an environmental exposure, and its relevance to human carcinogenesis remains to be established. Nevertheless, it shows that temporary disruption of chromatin repression can suffice to lastingly modify cell fate and to initiate tumorigenesis.

Most environmental perturbations are unlikely to produce such a complete and autonomous transition. More plausibly, they may create a latent susceptibility that becomes consequential only after later injury, inflammation, or genetic change. This raises the question of where such priming could persist over extended periods.

## Epigenetic memory in long-lived cells

### Long-lived cells as reservoirs of exposure memory

For an exposure-induced alteration to influence cancer years or decades later, it must be retained within a long-lived cell lineage. In rapidly renewing tissues, short-lived differentiated cells are unlikely to constitute this reservoir. Somatic stem cells, long-lived progenitors, and some differentiated cells with regenerative plasticity are more plausible candidates because they persist or generate descendants over extended periods. Consequently, the memory of exposure need not be present throughout a tissue: it may reside in a relatively small population and become apparent only during regeneration, inflammation, or another challenge (Fig. 2A and B).

Inflammatory memory provides an illustration of this process: following acute skin inflammation, mouse epithelial stem cells retained increased accessibility at selected stress-response loci and responded more rapidly to subsequent tissue injury [78]. Later work showed that AP-1 and tissue-specific transcription factors help establish and maintain these accessible “memory domains,” allowing rapid gene reactivation upon renewed challenge [79]. Such responses are initially adaptive because they accelerate tissue repair, but they demonstrate that a transient insult can durably alter the future behavior of a self-renewing population.

Long-term memory is not confined to canonical stem cells. In the mouse pancreas, after an episode of pancreatitis, acinar cells retained increased chromatin accessibility and H3K4me1 at metaplasia-associated genes [15]. This state altered their response to subsequent injury and lowered the threshold for transformation after activation of oncogenic K-Ras. The memory was reversible, as MAPK inhibition restored resistance to transformation [15]. Thus, a resolved inflammatory episode can leave a cancer-relevant chromatin alteration.

Another strong example comes from a mouse model of chronic colitis. Colonic stem cells retained altered chromatin accessibility and increased AP-1 activity for >100 days after inflammation had resolved [16]. Combined single-cell chromatin, transcriptomic, and lineage analyses showed that this memory was propagated cell-intrinsically through stem-cell divisions. When APC was subsequently deleted, previously inflamed tissues developed larger early tumors, and this advantage depended on AP-1 activity [16]. These studies show that a primed cell can remain apparently normal under homeostatic conditions while retaining an altered response to a later oncogenic event. Together, these studies and the external-exposure examples discussed above provide complementary evidence across the exposure–persistence–cancer pathway (Fig. 3B). Whether environmental chemicals can establish comparable cancer-relevant memories in defined long-lived mammalian cell populations remains less well established and represents an important direction for future research. These examples also illustrate that persistence need not imply faithful maintenance of the original chromatin alteration. A transient perturbation may establish an alternative regulatory or cellular state that persists even after the initiating molecular change has disappeared [80, 81]. It is therefore useful to distinguish persistence of a chromatin feature, persistence of the cell state that it helped establish, and persistence through selective expansion of an altered clone.

### Remaining silent during latency

In the models above, epigenetic memory altered the response to a later challenge without continuously driving proliferation (Fig. 2B). A primed cell may remain restrained by quiescence, its normal tissue niche, competition with neighboring cells, or the absence of a cooperating genetic alteration [82, 83].

Recent work on human hematopoietic stem cells illustrates this notion [84]. Following inflammatory stress, a subset of cells kept a transcriptional and epigenetic memory associated with quiescence and restrained hematopoietic output. Mutations associated with clonal hematopoiesis attenuated this restraint and promoted activation and differentiation [84]. Although this study did not establish an exposure-induced malignancy, it shows how inflammatory memory can coexist with long-term cellular quiescence and how genetic change may modify the subsequent behavior of the primed compartment.

The available experiments show persistence over weeks or months rather than decades, as in humans. Nevertheless, they support a model of latency as durable susceptibility rather than an occult tumor.

## From latency to clonal expansion

### Age, inflammation, and tissue ecology as selective pressures

Whether an epigenetically altered cell remains inconspicuous or expands may depend on its fitness relative to neighboring cells. A primed cell may remain rare while tissue architecture is intact and its competitors are healthy, but acquire an advantage when aging, injury, infection, inflammation, or repeated exposure alters the local environment [85–88]. Environmental exposures may contribute at this later stage by inducing chronic inflammation, metabolic stress, tissue damage, or niche remodeling that changes the relative fitness of primed and unprimed cells. These pressures may act positively, by promoting the survival or proliferation of primed cells, or negatively, by impairing their normal competitors [89]. Immune surveillance may likewise eliminate some altered cells while permitting immune-evasive or poorly recognized states to persist. Tissue ecology can therefore reshape clonal composition without requiring an exposure to induce the same stable chromatin alteration in every affected cell [90].

Clonal hematopoiesis illustrates how long latency and environmental selection can intersect, although the initiating events in this setting are usually genetic. Longitudinal analysis of human blood showed that hematopoietic clones can arise decades before detection and that their growth trajectories depend on both the driver mutation and age-related changes in the competitive environment [85]. In mouse models, chronic infection selectively expanded Dnmt3a-deficient hematopoietic stem cells through interferon-γ signaling, whereas inflammatory stimuli promoted the expansion and further genetic evolution of Trp53-mutant hematopoietic progenitors [86, 87]. These studies do not demonstrate environmentally induced epigenetic initiation, but they show that physiological and inflammatory conditions can determine whether an altered stem-cell clone remains inconspicuous or progresses toward malignancy.

Environmental exposures may therefore influence cancer at more than one stage: they may first perturb or prime a regulatory state and later provide the tissue context in which that state acquires a selective advantage. Aging, inflammation, tissue injury, or repeated exposure may thus determine whether a primed cell remains rare or undergoes clonal expansion.

### Progression and therapy response may also be influenced by earlier priming

Exposure-induced chromatin perturbations may influence not only cancer initiation but also subsequent progression and therapy response. Tumors are heterogeneous ecosystems in which metabolic stress, inflammation, hypoxia, immune pressure, and treatment continuously reshape regulatory states. An earlier priming event that increases cellular plasticity, stress tolerance, immune evasion, or access to drug-resistant states could therefore influence tumor evolution or relapse, even if it was not sufficient to initiate the cancer [91]. Alternatively, environmental conditions encountered after transformation may generate similar chromatin programs *de novo* or selectively expand cells in which they are already present.

These possibilities complicate causal interpretation. A chromatin state detected in an established tumor may descend from an earlier primed progenitor, may have been extensively remodeled or selected during progression, or may have arisen only after transformation. Demonstrating that an exposure-associated state promotes progression or treatment resistance therefore does not establish that the exposure initiated the cancer. Evidence for continuity with earlier priming is strongest when a regulatory potential can be followed across exposure withdrawal, transformation, and subsequent tumor evolution.

These uncertainties point to a broader challenge: establishing causality requires not only identifying an association between exposure, chromatin state, and cancer phenotype, but also reconstructing their temporal sequence. For cancers that develop over years or decades, it is particularly important to determine when an altered regulatory state arose, whether it persisted after exposure ended, in which cells it was retained, and when it became functionally relevant.

## Establishing causality over time

### Association is a starting point, not an endpoint

Human studies are essential for identifying chromatin signatures associated with real-world exposures, but they rarely establish causality on their own. Exposure-associated DNA methylation signatures, such as those observed after particulate matter or tobacco exposure [92–94], demonstrate the power of human epigenetic epidemiology, but also illustrate its limits. In many studies, it remains difficult to determine whether the observed chromatin changes occur in the relevant cell type, persist over time, reflect direct effects of exposure, or instead arise secondarily from inflammation, altered proliferation, immune infiltration, or changes in tissue composition [95].

An altered epigenome signature after exposure should therefore be interpreted as a hypothesis-generating observation. It may identify a relevant tissue, pathway, or window of susceptibility, but additional evidence is needed to determine whether the chromatin change contributes to malignant transformation. Nevertheless, an exposure-associated mark may be valuable as a biomarker even if it is not itself causal. Distinguishing biomarkers of exposure from mediators of cancer biology is a central challenge for the field.

### Going from association to a temporal mechanism

A convincing cancer-relevant epimutagen should connect exposure to phenotype through a testable molecular chain: exposure, proximal target or physiological mediator, chromatin alteration, gene-regulatory consequence, and cancer-relevant cellular phenotype. For diseases with long latency, this chain must be extended to include persistence after exposure withdrawal, residence within an appropriate long-lived cell population, a period of latency, and a later event that reveals or selects the primed state.

Environmental health studies outside cancer epigenetics have shown that real-world exposure data can be combined with mixture reconstruction, cellular models, organoids, and animal experiments to test mechanistic hypotheses across biological scales [20]. Following this convincing example, a candidate exposure linked to aberrant DNA methylation in a human cohort could be tested in a relevant normal, stem/progenitor, or premalignant model at physiologically plausible doses. The resulting chromatin changes could then be examined for persistence after exposure withdrawal, reversibility by targeted intervention, dependence on a candidate enzyme or metabolite, and effects on transcriptional programs. Cancer relevance would require a further test: does the persistent state alter differentiation, regenerative behavior, clonal fitness, or susceptibility to a later genetic, inflammatory, or environmental challenge?

Establishing causality also requires distinguishing direct from indirect routes to chromatin disruption. As discussed above, some environmental agents may bind chromatin regulators directly, whereas others act through metabolism, redox state, inflammation, the microbiome, or altered cell composition. Chemoproteomics and thermal proteome profiling can identify candidate direct targets [96, 97], while transcriptomic and chromatin profiling can place an exposure within known regulatory pathways [98]. However, identification of a molecular target does not establish that it accounts for the biological effect of an exposure. Toxicants are often pleiotropic, and several direct and indirect pathways may act together. Target-identification approaches should therefore be combined with perturbation or rescue experiments that test whether a proposed mechanism is necessary, sufficient, or merely one contributor among several.

### Testing persistence in the relevant cells

The choice of model system is equally important. As discussed above, models should reflect whether an exposure acts directly on the susceptible cell or indirectly. For cancer initiation, models should also contain the long-lived cell population in which exposure history could plausibly be retained.

Time must be treated as an experimental variable. Acute high-dose exposure followed immediately by molecular profiling can reveal biochemical responses, but cannot establish epigenetic memory or latency. More informative designs include chronic low-dose exposure, removal of the agent, serial analysis during recovery, and a later challenge introduced only after the immediate response has subsided. Lineage tracing can determine whether a persistent state is transmitted within particular clones, while transplantation or organoid re-derivation can help distinguish cell-intrinsic memory from continued influence of the tissue environment.

Low-input and single-cell chromatin methods are especially valuable because rare primed, long-lived, or premalignant states may be obscured in bulk assays [99–101]. Single-cell measurements nevertheless provide only snapshots unless combined with lineage information or longitudinal sampling. Apparent persistence in repeated tissue samples could otherwise result from recurrent induction, altered cell composition, or selection of a pre-existing population rather than mitotic inheritance of an exposure-induced state.

The inflammatory-memory studies discussed above exemplify this approach by combining exposure withdrawal, analysis of the cells retaining memory, lineage-resolved measurements, and a later oncogenic challenge [15, 16].

### Evaluating evidence for cancer-relevant epimutagens

To summarize this section, evidence for a cancer-relevant environmental epimutagen can be evaluated through four questions. Is the chromatin change associated with exposure? Is the molecular route understood? Does the change persist after exposure withdrawal, and in which cells? Finally, does it alter cancer susceptibility or the response to a later oncogenic or environmental challenge?

This is a tall order, but no single study need establish the entire causal chain. Human cohorts can identify exposure signatures and susceptible windows, whereas controlled models, exposure-withdrawal experiments, lineage tracing, and perturbation or rescue studies can test mechanism, persistence, and functional relevance. Together, these approaches can distinguish biomarkers of exposure from chromatin perturbations that contribute to cancer biology.

## Consequences for prevention, biomarkers, and interception

The framework we outlined has practical implications even before the full causal chain from exposure to cancer has been established. It can guide exposure reduction, interpretation of chromatin biomarkers, and efforts to identify persistent susceptibility states that might eventually be intercepted.

### Prevention and risk stratification

Prevention is the most immediate implication. If an exposure establishes or reinforces a persistent state that increases susceptibility to later transformation, reducing that exposure may prevent both immediate damage and longer-term biological priming. Particular attention should be given to susceptible periods, including development, tissue injury, or regeneration, and chronic inflammatory or metabolic disease, when long-lived stem or progenitor populations may be especially responsive to environmental signals.

Exposure-associated chromatin changes may also provide useful biomarkers even when they are not themselves causal. They could record cumulative exposure, identify individuals or tissues that have responded unusually strongly, or reveal persistent states associated with increased susceptibility [12, 102].

The tissue in which a signature is measured is equally important. A blood-based mark may provide a useful record of systemic exposure without being present in the epithelial cell population from which a tumor later develops. Conversely, a biologically important alteration confined to a small target-cell population may be undetectable in bulk tissue. Biomarker studies should therefore distinguish explicitly among markers of exposure, markers of biological response, and markers of a persistent cancer-relevant state.

### Acting on persistent susceptibility states

A longer-term possibility is cancer interception: reversing a persistent primed state or preventing expansion of a susceptible clone before malignant progression. The pancreatic inflammatory-memory model provides proof of principle that a cancer-predisposing chromatin state can remain pharmacologically reversible, although intervention in people without overt disease would present substantial challenges [15].

In established cancers, drugs targeting DNA methylation, histone deacetylation, histone methylation, and related pathways can reprogram tumor cells or induce senescence [103–107]. However, activity in tumors should not be equated with safe prevention in otherwise healthy individuals. Chromatin regulators have broad functions in normal tissues, and prolonged systemic treatment could itself disrupt cell identity or tissue homeostasis.

Exposure reduction and control of chronic inflammation, metabolic dysfunction, or tissue injury are therefore more immediately realistic than treatment with broadly acting epigenetic drugs. More selective interception may become possible if a persistent susceptibility state can be traced to a defined, reversible pathway in a specific long-lived cell population. The central goal is to identify exposure-induced states that can be detected before cancer develops and, where possible, reversed safely through integration of epidemiology, toxicology, cancer epigenetics, and mechanistic models.

## Concluding remarks

Environmental epigenetics is most useful for cancer when framed not as a search for epimutations analogous to DNA mutations, but as the study of how exposures perturb chromatin-regulatory systems controlling cell identity, plasticity, and fitness. Most exposure-induced chromatin changes are likely to be transient, adaptive, or secondary. The central challenge is to identify those that become biologically embedded and contribute to cancer-relevant phenotypes.

Latency is critical to this question. An exposure-induced alteration need not remain molecularly unchanged for decades or be present in every exposed cell. Its lasting effect may instead reside in altered cellular potential retained within a long-lived cell population and revealed only when aging, inflammation, tissue injury, or a cooperating genetic alteration changes the cellular or selective context. It is therefore essential to distinguish persistence of a particular chromatin mark from persistence of a primed cell state or clone.

Establishing such mechanisms will require linking exposure to a molecular target or physiological mediator, chromatin alteration, gene-regulatory consequence, and phenotype. For diseases with prolonged latency, such as cancer, this chain must also establish persistence after exposure withdrawal, identify the cells that retain the altered potential, and determine which later events reveal or select it. Integrating epidemiology with physiologically realistic models, longitudinal and lineage-resolved analyses, single-cell profiling, chemical biology, and functional perturbation should help identify preventable exposures, informative biomarkers, and persistent susceptibility states that may eventually be intercepted.

## Data Availability

This is a review article. No new data were generated or analysed in support of this research.
